# Magnetocrystalline and Surface Anisotropy in CoFe_2_O_4_ Nanoparticles

**DOI:** 10.3390/nano10071288

**Published:** 2020-06-30

**Authors:** Alexander Omelyanchik, María Salvador, Franco D’Orazio, Valentina Mameli, Carla Cannas, Dino Fiorani, Anna Musinu, Montserrat Rivas, Valeria Rodionova, Gaspare Varvaro, Davide Peddis

**Affiliations:** 1 Institute of Structure of Matter–CNR, Monterotondo Stazione, 00016 Rome, Italy; asomelyanchik@kantiana.ru (A.O.); salvadormaria@uniovi.es (M.S.); Dino.Fiorani@ism.cnr.it (D.F.); gaspare.varvaro@ism.cnr.it (G.V.); 2 Institute of Physics, Mathematics and Information Technology, Immanuel Kant Baltic Federal University, 236041 Kaliningrad, Russia; vvrodionova@kantiana.ru; 3 Department of Physics, University of Oviedo, 33204 Gijón, Spain; mrivasardisana@gmail.com; 4 The Department of Physical and Chemical Science, University of L’Aquila, Via Vetoio, Coppito, 67100 L’Aquila, Italy; franco.dorazio@aquila.infn.it; 5 Department of Geological and Chemical Sciences, University of Cagliari, Cittadella Universitaria, 09042 Monserrato, Italy; valentina.mameli@unica.it (V.M.); ccannas@unica.it (C.C.); musinu@unica.it (A.M.); 6 National Interuniversity Consortium of Materials Science and Technology (INSTM), Via Giuseppe Giusti 9, 50121 Firenze, Italy; 7 Department of Chemistry and Industrial Chemistry (DCIC), University of Genova, 16146 Genova, Italy

**Keywords:** magnetic nanoparticles, cobalt ferrite, magnetic anisotropy

## Abstract

The effect of the annealing temperature *T*_ann_ on the magnetic properties of cobalt ferrite nanoparticles embedded in an amorphous silica matrix (CoFe_2_O_4_/SiO_2_), synthesized by a sol-gel auto-combustion method, was investigated by magnetization and AC susceptibility measurements. For samples with 15% w/w nanoparticle concentration, the particle size increases from ~2.5 to ~7 nm, increasing *T*_ann_ from 700 to 900 °C. The effective magnetic anisotropy constant (*K*_eff_) increases with decreasing *T*_ann_, due to the increase in the surface contribution. For a 5% w/w sample annealed at 900 °C, *K*_eff_ is much larger (1.7 × 10^6^ J/m^3^) than that of the 15% w/w sample (7.5 × 10^5^ J/m^3^) annealed at 700 °C and showing comparable particle size. This indicates that the effect of the annealing temperature on the anisotropy is not only the control of the particle size but also on the core structure (i.e., cation distribution between the two spinel sublattices and degree of spin canting), strongly affecting the magnetocrystalline anisotropy. The results provide evidence that the magnetic anisotropy comes from a complex balance between core and surface contributions that can be controlled by thermal treatments.

## 1. Introduction

Within the last few years, magnetic nanoparticles have contributed to the development of a variety of cutting edge technologies in fields such as ferrofluids [1], microwave devices [2], biomedicine [3,4], or catalysis [5,6]. The growing interest that magnetic nanoparticles attract demands a fundamental understanding of their properties, which are very different from their bulk counterparts. In this context, spinel ferrites are excellent candidates thanks to their tunable physico-chemical properties [7]. Their general chemical formula is MFe_2_O_4_, where M^2+^ can be any divalent metal (e.g., M^2+^ = Fe^2+^, Co^2+^, Zn^2+^, Ni^2+^, Mn^2+^, etc.). The atomic arrangement corresponds to a face-centered cubic structure of the oxygen atoms, with Fe^3+^ and M^2+^ occupying the tetrahedral (*T_d_*) and octahedral (*O_h_*) sites [7]. Such a structure makes magnetic spinel nanoparticles particularly attractive. It provides a tool to tailor their magnetic properties (e.g., magnetic crystalline anisotropy and saturation magnetization) by the variation of the cation distribution between the two sublattices. This can be done by changing the chemical composition, the preparation method, and thermal treatments [8,9,10].

Magnetic properties of spinel ferrite nanoparticles are also strongly affected by the presence of a non-collinear spin structure (i.e., spin canting). The spin-canting is due to competing interactions between sublattices [11,12], as confirmed by polarized neutron scattering [13] and ^57^Fe Mössbauer experiments [14,15]. This symmetry breaking induces changes in the topology of the surface magnetic moments and, consequently, in the exchange integrals (through super-exchange angles and/or distances between moments), thus leading to a change in the surface anisotropy [15]. Therefore, the magnetic properties of ferrite nanoparticles with a spinel structure are due to a complex interplay of several effects, among which surface disorder, cationic distribution, and spin canting are dominant [14,16].

The present work is aimed at investigating the effect of the annealing temperature on the magnetic properties of nanocomposites consisting of CoFe_2_O_4_ nanoparticles dispersed in a silica matrix (CoFe_2_O_4_/SiO_2_). The results show that the thermal treatment plays an important role, along with the particle size, in controlling the surface and core contributions to the magnetic anisotropy and saturation magnetization.

## 2. Materials and Methods 

A set of CoFe_2_O_4_ nanoparticles uniformly embedded in a silica matrix with 15% (w/w) concentration of the magnetic phase were synthesized by a sol-gel auto-combustion method and treated afterward at three different annealing temperatures (T_ann_ = 700, 800 and 900 °C). Synthesis and morpho-structural characterization of all the samples was already described in detail elsewhere [8,14,17,18] 

The Fe(NO_3_)_3_∙9H_2_O (Sigma Aldrich 98%, Darmstadt, Germany), Co(NO_3_)_2_∙6H_2_O (Sigma Aldrich 98%, Darmstadt, Germany), citric acid (Sigma Aldrich 99.5%, Darmstadt, Germany) and of 25% ammonia solution (Carlo Erba Reagenti SpA, Cornaredo, Italy) were used without further purification. In this process, 1-molar iron and cobalt nitrate aqueous solutions in a 2:1 ratio, respectively, and citric acid (CA) with 1:1 molar ratio of metals to CA were prepared, and pH-adjusted to ~2 by aqueous ammonia addition. Tetraethoxysilane (TEOS, Sigma Aldrich 98%, Darmstadt, Germany) in ethanol was used as a silica precursor and, after its addition and vigorous stirring for 30 min, the sols were placed in an oven to gel in static air at 40 °C for 24 h. The gels underwent successively a thermal treatment at 300 °C for 15 min, where the auto-combustion reaction took place.

The temperature was then raised to 900 °C in steps of 100 °C and kept for 1 h at the treatment temperature. The X-ray diffraction (XRD) patterns [18] (Figure S1, reported in supporting materials) show a big halo due to the amorphous silica; the main reflections due to the cubic cobalt ferrite phase start to appear at 700 °C, and they become more and more evident at 800 and 900 °C [18]. For this reason, investigation of the magnetic properties was focused on samples treated at 700, 800 and 900 °C, hereafter named N15T700, N15T800, and N15T900. Transmission electron microscopy (TEM) (Figure S2, reported in supporting materials) shows the presence of crystalline particles for all the samples. The heating process led to the progressive growth of the particles and their structural ordering. The high resolution TEM images performed on N15T900 (Figure S2, Supplementary Materials) confirm the particles’ spherical morphology. The observed set of fringes corresponds to the (311) lattice planes of the cobalt ferrite phase with a distance of 2.4 Å.

The particle size distribution obtained by TEM image analysis can be fitted by a log-normal function [19]:

(1) P=ADσ2πexp−[ln2(D〈DTEM〉)2σ2]

where <*D*_TEM_> is the median of the variable “*diameter*” (Table 1) and *σ* is the standard deviation. An increase in particle size with the increase in annealing temperature is observed. 

DC-magnetization measurements were performed using a SQUID magnetometer (Quantum Design Inc., San Diego, CA, USA) equipped with a superconducting magnet producing fields up to 5 T. AC-susceptibility measurements were performed at different frequencies (20–800 Hz) as a function of the temperature using a susceptometer (Model ACS 7000, Lake Shore Cryotronics Inc., Weterville, OH, USA).

## 3. Results and Discussions

**Figure 1:**
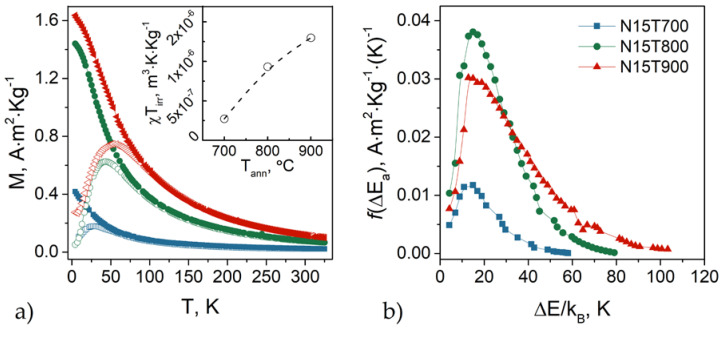
(**a**) Zero-field-cooled (ZFC) (empty symbols) and field-cooled (FC) (solid symbols) magnetization curves. Inset: product of the magnetic susceptibility times the irreversibility temperature as a function of the annealing temperature. (**b**) Energy barrier distribution obtained from the first derivative of the thermoremanent magnetization *M*_TRM_(*T*) versus temperature.

**Figure 2:**
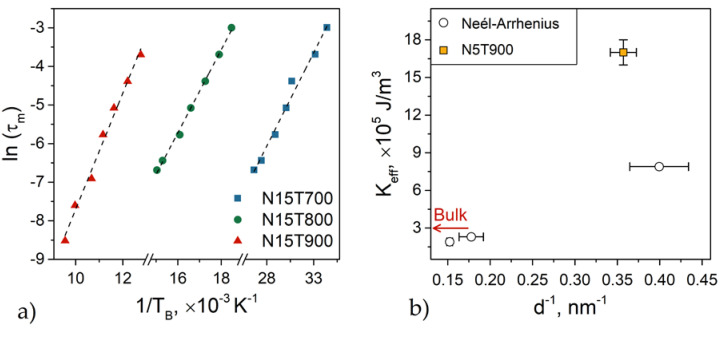
(**a**) Logarithm of the measurement time $\tau_{m}$ versus $1/T_{B}$ and its linear fit (dashed line); (**b**) effective anisotropy constant *K*_eff_ of N15T700, N15T800 and N15T900 obtained from fitting $ln\left(\tau_{m}\right)$ versus $1/T_{B}$ by Neél–Arrhenius model (empty circles). The value from N5T900 was taken from reference [14] and that of bulk cobalt ferrite from reference [7].

The temperature dependence of the zero-field-cooled/field-cooled (ZFC/FC) magnetizations is shown in Figure 1a. The sample was cooled down to 4.2 K from room temperature in the absence of an applied field. Then, the ZFC curve was recorded in a field of 5 mT while warming up to 325 K. In contrast, the FC curve was recorded after having cooled the sample down (from 325 to 4.2 K) with the same field applied. The shape of the FC-curves suggests that interparticle interactions are negligible [19,20,21]. The temperature corresponding to the maximum in the ZFC curve, *T*_max_, (Table 1) increases with the annealing temperature. According to Gittleman et al. [22], *T*_max_ is related to the average blocking temperature <T_B_> through the equation:

(2) $$T_{\max}\approx \;\beta <T_{B}>$$

where *β* is a constant that, for a log-normal distribution of particle sizes, is in the range of 1.5–2.5. The temperature at which the ZFC and FC curves merge is the irreversibility temperature (*T*_irr_), and it corresponds to the blocking temperature of the particles with the maximum anisotropy. As expected, both *T*_irr_ and *T*_max_ grow with increasing size (i.e., increasing temperature). The difference between *T*_irr_ and *T*_max_ reflects the width of the blocking temperature distribution in the absence of magnetic interparticle interactions and it is correlated to the volume distribution. In our samples, such difference is weakly dependent on the annealing temperature, indicating that the thermal treatment does not significantly affect the distribution of the blocking temperatures. This is confirmed by the thermoremanent magnetization (TRM) curves [21] (Figure 2b, see supplementary information for details). Indeed, the shape of the energy barrier distribution is similar for the three samples, confirming that the sources of anisotropy are basically the same and that the interparticle interactions are weak. Two different models have been proposed to determine the blocking temperature distribution, yielding to its mean value and standard deviation. Starting from the model proposed by Chantrell and co-workers [23], the distribution of the anisotropy energy barriers was fitted by a log-normal function to determine the mean value of the blocking temperature (*<T_B_>_CH_*), reported in Table 1 [24,25,26]. We give details of the fit and values of the standard deviation (*σ*_TRM_) in the supporting information (Figures S3 and S4).

Hansen and Mørup proposed a phenomenological approach to calculate the mean blocking temperature (<*T_B_*>_H.M._) and its standard deviation (*σ*_H.M._) [27] for a log-normal distribution of the particles volume, and negligible interparticle interactions. They found that <*T_B_*>_H.M._ and *σ*_H.M_ can be expressed with known values of *T*_irr_ and *T*_max_ from *<T_B_>*_H.M._ = *T*_max_ [1.792 + 0.186·ln(*T*_irr_/*T*_max_ − 0.918)]^−1^ + 0.0039*·T*_irr_ and *σ*_H.M._ = 0.624 + 0.397 ln(*T*_irr_/*T*_max_ − 0.665). <*T_B_*>_H.M._ values are given in 16(1), 25(2) and 31(2) K for samples (Table 1), their standard deviation values being 0.73, 0.61, and 0.57 for N15T700, N15T800 and N15T900, respectively. 

The values of the mean blocking temperatures extracted by the two models are equal within the experimental errors (Table 1). The percentual polydispersity of the blocking temperatures is defined as:

(3) $$PD\%=100\times \frac{\sigma }{\langle T_{B}\rangle }$$

The *PD*% value obtained for Chantrell and Hansen–Mørup models (*PD*_CH._ and *PD*_H.M._) decreases with increasing particle size, although this trend is more evident for the Chantrell model.

The inset in Figure 1a shows the product of the susceptibility times the irreversibility temperature (*χT*_irr_) as a function of the annealing temperature (*T*_ann_). These results indicate a strong increase in the ferrimagnetic phase between 700 and 900 °C, which can be ascribed to the rise in the particle volume [28].

The dynamic magnetic properties were investigated by AC-susceptibility measurements in a field of 2.5 mT at frequencies *υ* from 5 Hz to 10 kHz, in the temperature interval 18–310 K. According to the Néel–Arrhenius model, the relaxation process of the particle moments is driven by thermal activation and described, in the absence of interparticle interactions, by the Arrhenius law *τ_N_ = τ*_0_ exp(*K*_eff_*V*/*k_B_T*). Since *T* = *T_B_* when *τ_m_ =* 1/*υ*_m_, a linear relation between *ln(τm)* and *1/T_B_* can be derived:

(4) $$ln(\tau_{m})=ln(\tau_{0})+\frac{K_{\mathrm{eff}}V}{k_{B}T_{B}}.$$

In Figure 2a, the linear relationship between *ln(τm)* versus *1/T_B_* is reported for the three samples. The values of the effective magnetic anisotropy constant, *K*_eff_, and the characteristic relaxation times, *τ*_0_, obtained from the linear fitting of Equation (4), are given in Table 2.

For the sample annealed at the lowest temperature (N15T700), the *τ*_0_ value has a coherent physical meaning (1.9 × 10^−9^ s), confirming the absence of interparticle interactions. On the other hand, for samples N15T800 and N15T900, the *τ*_0_ value is much smaller. This fact indicates that the Néel–Arrhenius model is not appropriate to describe the dynamical behavior of these samples, suggesting that weak interparticle interactions are present.

According to the Vogel–Fulcher law, weak interparticle interactions are accounted for by a temperature term *T*_0_ [29,30,31]:

(5) $$ln(\tau_{m})=ln(\tau_{0})+\frac{K_{eff}V}{k_{B}\left(T_{B}+T_{0}\right)}.$$

The values of *T*_0_ and *K*_eff_ (Table 2) have been obtained from the fitting of Equation (5) by fixing the specific relaxation time *τ*_0_ equal to 10^−10^ s for all the samples [14,26]. In sample N15T700, *T*_0_ is almost zero, consistent with the absence of interparticle interactions. Then, *T*_0_ rises with the annealing temperature, indicating an increase in the dipolar interactions due to the enhancement of the particle magnetic moment. It is worth underlining that the value of *K*_eff_ obtained by Néel–Arrhenius and Vogel–Fulcher models are similar for sample N15T700 where the interactions can be considered negligible. A difference in the *K*_eff_ values deduced from the two models is observed for N15T800 and N15T900 due to magnetic interactions.

On the other hand, in both models, *K*_eff_ increases with a decreasing particle size (i.e., decreasing annealing temperature). We measured a rise of ~30% when the diameter goes from 6.6 (N15T900) to 5.6 nm (N15T800), while a much higher growth of ~70% is observed when it goes from 6.6 (N15T800) to 2.5 nm (N15T700). This result indicates that the surface anisotropy increases with a decrease in the particle size, but its role becomes dominant in tiny particles (e.g., N15T700). This idea is also confirmed by the fact that the *K*_eff_ values of N15T800 and N15T900, which are lower than the value of the bulk material (3 × 10^5^ J/m^3^ [14,32]), indicating that the magnetic structure also plays a crucial role. The smaller anisotropy in CoFe_2_O_4_ nanoparticles compared to the bulk value can be related to a change in the cation distribution with the size, induced by the annealing treatment. This phenomenon was already observed in CoFe_2_O_4_ particles [14,33] and explained by a modification of the cation distribution leading to a change in the magneto-crystalline anisotropy mainly determined by the distribution of the Co^2+^ ions between *O_h_* and *T_d_* sites. Indeed, here the cause can be a lower fraction of Co^2+^ ions in the octahedral sites, having larger anisotropy (+850 × 10^−24^ J/ion) (due to the orbital contribution in the crystal field ^4^T_1_ ground energy term) than Co^2+^ ions in a tetrahedral site (−79 × 10^−24^ J/ion; ^4^A_2_ term) [8].

To highlight these results, Figure 2b reports the *K*_eff_ value of an additional sample consisting of CoFe_2_O_4_ nanoparticles embedded in a silica matrix with a 5% (w/w) concentration of magnetic phase annealed at 900 °C (hereafter named N5T900). For this sample, the average particle size (2.8 ± 0.3 nm [14]) is very close to that of the N15T700 (2.5 ± 0.5 nm), with the same percentual polydispersity (see Figure S5 and Table S1 in the supporting information). It is important to underline that the interparticle interactions in both N5T900 and N15T700 samples are negligible, as indicated by their corresponding ZFC-FC and *δM*-plots [34] (Figures S6 and S7, respectively, in the supporting information).

Despite the two samples having the same morphological features, *K*_eff_ is much larger for N5T900, which could be related to the cation distribution change caused by the annealing. The highest temperature produces a larger occupancy of *O_h_* sites by the Co^2+^ ions in sample N5T900 [14].

**Figure 3:**
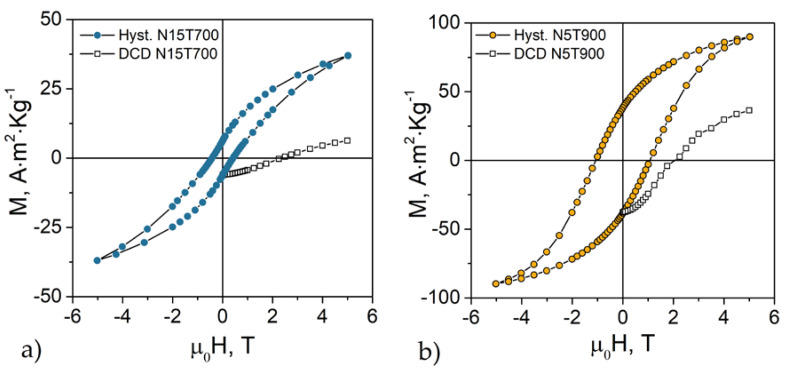
Field-dependence of magnetization and direct current demagnetization (DCD) curves measured at 5 K for (**a**) sample N15T700 and (**b**) reference sample N5T900 with the same particle size [14].

Low-temperature (5 K) magnetization loops of the samples N15T700 and N5T900 are reported in Figure 3. They are not saturated due to their high anisotropy (the same as samples N15T800 and N15T900, reported in Figure S8 of the supporting information). The saturation magnetization (*M*_S_) has been estimated by fitting the high field range of the curves to the equation [35]:

(6) $$M\left(H\right)=M_{S}\cdot \left(1-\frac{a}{H}-\frac{b}{H^{2}}\right)+H\cdot \chi_{\mathrm{SAT}}$$

where *a* and *b* are the fitting parameters, and *χ*_SAT_ is the “non-saturated” magnetic susceptibility (for high applied fields). The latter is strongly related to the non-collinear spin structure due to competing interactions between sublattices, and to the symmetry breaking at the particle surface [36,37].

**Figure 4:**
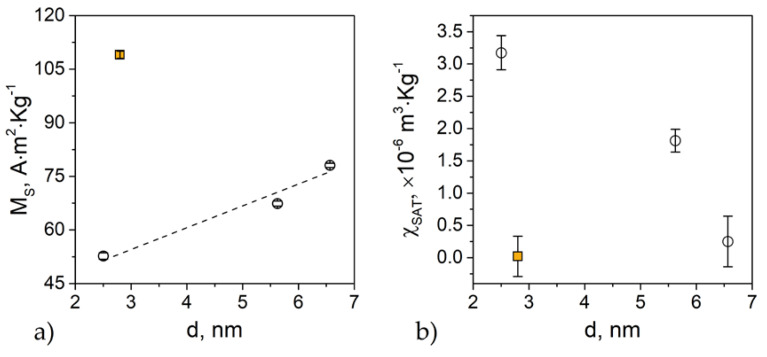
(**a**) Saturation magnetization, $M_{S}$, and (**b**) non-saturated susceptibility, $\chi_{\mathrm{SAT}}$, obtained by fitting Equation (6). The dashed line represents a guide for the eye. The square in both graphs corresponds to the reference sample N5T900.

Figure 4a shows that *M_S_* increases with particle size (i.e., annealing temperature), as expected. In the same figure, we plot *M_S_* for sample N5T900 (2.8 ± 0.3 nm particle size). Despite N5T900 and N15T700 having the same particle size, *M_S_* for N5T900 is almost twice than for N15T700. Considering that the magnetic interparticle interactions are negligible in both samples, this difference can be ascribed to the combined effect of cation distribution, spin-canting, and surface anisotropy [14,16]. The non-saturated susceptibility (Figure 4b) increases with decreasing particle size (i.e., decreasing annealing temperature) [31]. The trend of *χ*_SAT_ indicates, as expected, the more substantial contribution of the surface magnetic anisotropy for smaller particles. It is worth emphasizing that N15T700 has a higher value of *χ*_SAT_, indicating that the surface contribution to the effective magnetic anisotropy is higher in N15T700 than in N15T900. The energy barrier distribution can confirm this. In fact, despite N5T900 and N15T700 having the same *PD*% of the TEM diameter, the *PD*% for *T_B_* calculated by H.M. model is much higher for N15T700 (*PD*% *T_B_* 4.56) than for N5T900 (*PD*% *T_B_* 2.45).

Given that the particle volume is comparable in the two samples, this fact can be explained by the mentioned surface anisotropy contribution. Labarta and co-workers have shown that, for spinel ferrite nanoparticles, even when the size distribution is narrow, surface anisotropy can produce a substantial broadening of the anisotropy energy distribution. This effect is an obvious consequence of the different size dependence of the energy contributions from the core and the surface [38]. Because the volume content of the surface spin layer increases with a decrease in size, and it becomes more significant for ultra-small particles (<10 nm). 

Then, although *χ*_SAT_ and the anisotropy energy barrier distribution indicate a more significant contribution of the surface component to the anisotropy in N15T700, the value *K*_eff_ obtained by AC measurements is higher in N5T900. This could be associated with an increase in the magneto-crystalline component of the anisotropy.

The squares in Figure 3 represent the low temperature (5 K) direct current demagnetization (DCD) remanent curves measured following the protocol in ref. [39]: (1) application of a quasi-saturating field of 5 T; (2) application of a gradually increasing magnetic field in the reversal direction; (3) switching off the magnetic field and collection of the remanent magnetization value after each iteration. Since each measurement is performed at zero field, *M*_DCD_ is only sensitive to the irreversible component of the magnetization and only the blocked particles contribute to the remanent magnetization. The curve shape is linked to the switching field distribution, which, in turn, is related to the energy barrier distribution; the value of the field at which the remanent magnetization is equal to zero (called remanent coercivity, *H*_Cr_) corresponds to the mean switching field. Although the two samples have different coercivity, the remanent coercivities are close (N15T700: ~2.4 T, N5T900: ~2.1 T). This result is in line with the similar anisotropy fields (N15T700: 5.8(5) T, N5T900: 5.9(6) T) estimated by the Stoner–Wohlfarth model (*H_K_* = 2*K*_eff_*/M_S_*).

Even though *H*_Cr_ and *H*_K_ are equal within the experimental error, the coercivities of the two samples are different. Since both systems are non-interacting, such differences can be associated with a larger fraction of very small particles that probably are not well crystallized due to the low treatment temperature. We have confirmed this by the trend of *χT*_irr_ (inset of Figure 1a) and the lower value of the remanent and saturation magnetizations [40].

## 4. Conclusions

The results provide evidence that the relative surface and core contributions to the effective magnetic anisotropy and saturation magnetization of CoFe_2_O_4_ nanoparticles embedded in a silica matrix can be controlled by the annealing temperature *T*_ann_. For samples with 15% w/w of nanoparticles, the value of the effective magnetic anisotropy constant *K*_eff_ increases and the saturation magnetization decreases by decreasing *T*_ann_ from 900 to 700 °C, with a decrease in particle size, showing a dominant role of the disordered surface. On the other hand, the comparison between the 15% w/w sample annealed at 700 °C and a 5% w/w sample annealed at 900 °C, with comparable particle size, (2.5 and 2.8 nm with the same size distribution) shows a much larger saturation magnetization and *K*_eff_ values for the latter one, for which the *χ*_SAT_ value, related to non-collinear core spin structure and surface disorder, is much lower. The comparison indicates that for the 5% w/w with *T*_ann_ = 900 °C sample the major contribution to the anisotropy comes from the core, despite its very small particle size. This should be due to a better crystallinity and change in a core structure (e.g., different cation distribution and degree of spin canting) with larger magnetocrystalline anisotropy induced by the higher *T*_ann_. In conclusion, the results indicate that the effect of the annealing temperature on the anisotropy and saturation magnetization is not limited to the change in the particle size, increasing with *T*_ann_. Besides the decrease in surface disorder, the core structure is also affected by the thermal treatment, which can significantly modify the magnetocrystalline anisotropy and the saturation magnetization.

## Figures and Tables

**Table 1:** Structural and magnetic properties.

Sample	*d*_TEM_	*T*_max_	*T*_irr_	*<T_B_>_CH._* ^2^	*PD*_CH._	<*T_B_*>_H.M._^2^	*PD*_H.M._
	(nm)	(K)	(K)	(K)	(%)	(K)	(%)
N15T700	2.5(2) ^1^	29(1)	57(5)	18(1)	3.26	16(1)	4.56
N15T800	5.3(5)	43(1)	70(5)	22(2)	2.86	25(2)	2.44
N15T900	6.6(5)	53(1)	82(3)	29(1)	2.41	31(2)	2.45

^1^ Uncertainties in the last digits are given in parenthesis; ^2^ Average blocking temperature extracted from thermoremanent magnetization (TRM) (<*T_B_*>_CH._) and (<*T_B_*>_H.M._) from Hansen and Mørup method are reported with their corresponding percentual polydispersity index.

**Table 2:** Magnetic parameters obtained from AC magnetic susceptibility measurements.

Sample	Néel−Arrhenius ^1^		Vogel−Fulcher ^2^	
	$K_{eff}\;$	$\tau_{0}\;$	$K_{eff}\;$	$T_{0}$
	(J m^−3^)	(s)	(J m^−3^)	(K)
N15T700	7.9(4) × 10^5^	1.9 × 10^−9^	11(1) × 10^5^	−1(3)
N15T800	2.3(2) × 10^5^	8.2 × 10^−14^	1.3(1) × 10^5^	14(2)
N15T900	1.9(2) × 10^5^	1.5 × 10^−14^	0.92(1) × 10^5^	32(3)

^1^ Effective magnetic anisotropy constant (*K*_eff_) and characteristic relaxation time (*τ*_0_) obtained from the fitting to Néel−Arrhenius law (Equation (4)); ^2^ *K*_eff_ and the interaction temperature term, *T*_0_, assuming *τ*_0_ = 10^−10^ s, from Vogel−Fulcher law (Equation (5)).

## Supplementary Materials

The following are available online at https://www.mdpi.com/2079-4991/10/7/1288/s1, Figure S1: XRD patterns of CoFe2O4 nanoparticles embedded (15% w/w) in an amorphous silica matrix and annealed at different temperatures (from ref. [18]); Figure S2: TEM image of as-prepared sample (a); dark field images of N15T700 (b) and N15T800 (c); bright field (d) and high resolution image of N15T900 (from ref. [18]); Figure S3: TRM measurements (full symbols) and corresponding distribution of magnetic anisotropy (empty symbols) energies with cooling field of 2.5 mT for the samples N15T700 (a), N15T800 (b), N15T900 (c); Figure S4: distribution of magnetic anisotropy energies (empty symbols) fitted by a log normal function (lines) for the samples N15T700 (squares), N15T800 (triangles), N15T900 (circles). Values of mean Blocking temperature (<T_B_>) and standard deviation (σ) are reported in the graph; Figure S5: Particle size distribution extracted by TEM images of the sample N15T700 (left side) and N5T900 (right side); Figure S6: ZFC-FC curves for the N15T700 (a) and N5T900 (b); Figure S7: δM-plot at 5 K for N15T700 sample and reference sample N5T900 [14]; Figure S8: Hysteresis loop recorded at 5 K for N15T900 (a), N15T800 (b) and N15T700 (c); Table S1: Mean particle size (*D*_TEM_) σ the standard deviation of the natural logarithm of the variable D and percentual polydispersity determined by Equation (7).
