# Supplementary material for: Magnetocrystalline and Surface Anisotropy in CoFe2O4 Nanoparticles
Source: Nanomaterials (Basel). 2020 Jun 30;10(7):1288. doi: 10.3390/nano10071288 (PMC7408426; doi:10.3390/nano10071288)
Supplement: Supplementary file 1 [file nanomaterials-10-01288-s001.pdf]

nanomaterials

Article

Magnetocrystalline and Surface Anisotropy in $CoFe_2O_4$ Nanoparticles

Alexander Omelyanchik [1,2], María Salvador [1,3], Franco D'Orazio [4], Valentina Mameli [5,6], Carla Cannas [5,6], Dino Fiorani [1], Anna Musinu [5,6], Montserrat Rivas [3], Valeria Rodionova [2], Gaspare Varvaro [1] and Davide Peddis [1,6,7,*]

1 Institute of Structure of Matter–CNR, 00016, Monterotondo Stazione, Rome, Italy; asomelyanchik@kantiana.ru (A.O.); salvadormaria@uniovi.es (M.S.); Dino.Fiorani@ism.cnr.it (D.F.), gaspare.varvaro@ism.cnr.it (G.V.)
2 Immanuel Kant Baltic Federal University, 236041, Kaliningrad, Russia; vvrodionova@kantiana.ru
3 Department of Physics, University of Oviedo, 33204, Gijón, Spain; salvadormaria@uniovi.es (M.S.), mrivasardisana@gmail.com (M.R.)
4 The Department of Physical and Chemical Science, University of L'Aquila, Via Vetoio, Coppito, L'Aquila 67100, Italy; franco.dorazio@aquila.infn.it
5 Department of Geological and Chemical Sciences, University of Cagliari, Cittadella Universitaria, 09042, Monserrato, Italy; valentina.mameli@unica.it (V.M.), ccannas@unica.it (C.C.), musinu@unica.it (A.M.)
6 National Interuniversity Consortium of Materials Science and Technology (INSTM), Via Giuseppe Giusti 9, Firenze, 50121, Italy
7 Department of Chemistry and Industrial Chemistry (DCIC), University of Genova, 16146, Genova, Italy
* Correspondence: davide.peddis@unige.it; Tel.: +39-010-776-7974

SUPPORTING INFORMATION

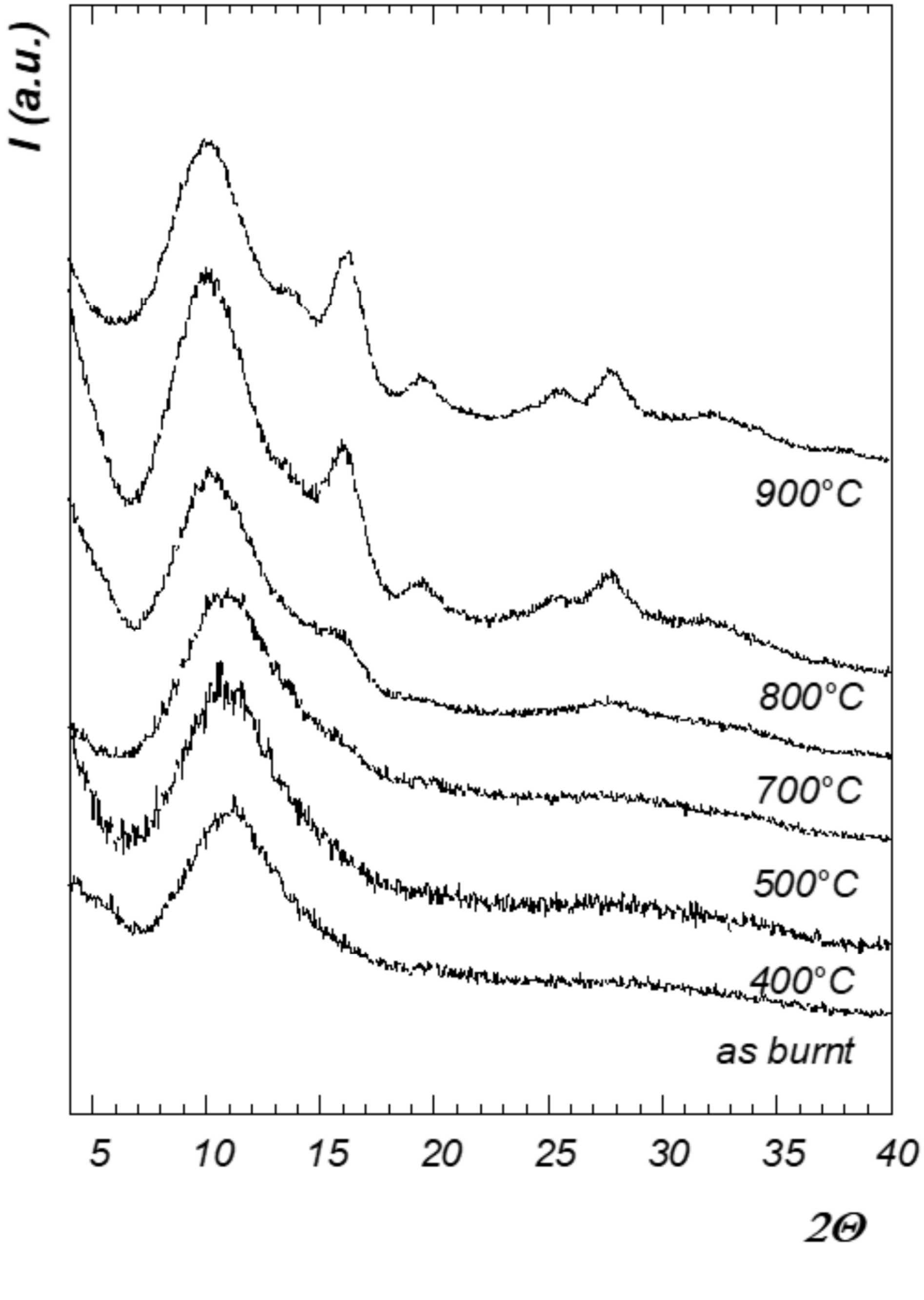

Figure S1. XRD patterns of $CoFe_2O_4$ nanoparticles embedded (15% w/w) in an amorphous silica matrix and annealed at different temperatures (from ref.[1]).

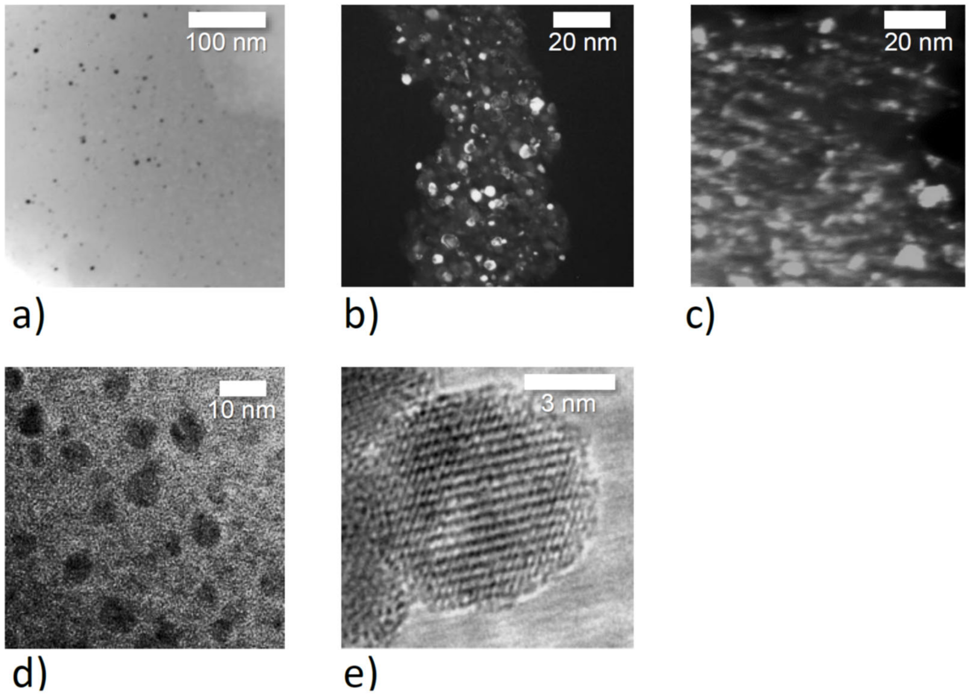

Figure S2. TEM image of the as-prepared sample (a); dark field images of N15T700 (b) and N15T800 (c); bright field (d) and high resolution image of N15T900 (from ref.[1]).

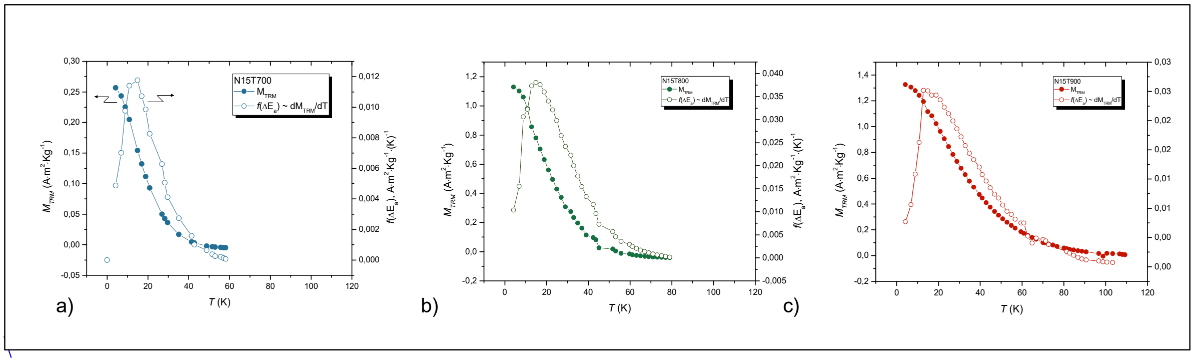

Figure S3. TRM measurements (full symbols) and corresponding distribution of magnetic anisotropy (empty symbols) energies with cooling field of 2.5 mT for the samples N15T700 (a), N15T800 (b), N15T900 (c).

In thermoremanent magnetization (TRM) measurements the samples have been cooled down from 300 K in an external static magnetic field of 2.5 mT. At 5 K the field is then turned of, and the remanent magnetization was measured on warming up. For an assembly of noninteracting particles, M_{TRM} is related to the distribution of anisotropy energy barriers:

$$M_{TRM} = M_{nr} \int_{\Delta E_c}^{\infty} f(\Delta E_a)\, dE \qquad (1)$$

where M_{nr} is the non-relaxing component of the magnetization and ΔE_c is a critical value of energy, above which all the particles are blocked. The presence of an inferior limit (ΔE_c) in the integral means that, at a given temperature and for a given applied field, only the particles for which the energy barrier is higher than ΔE_c contribute to the measured M_{TRM}. From equation 1 is clear that the temperature derivative of MTRM provides an estimation of the energy barrier distribution.

$$f(\Delta E_a) \sim \frac{dM_{\mathrm{TRM}}}{dT} \qquad (2)$$

Considering particles with uniaxial anisotropy and temperature independent anisotropy constant $\Delta Ea = KV \propto T_B$ and equation (2) can re-written as

$$f(T_B) \sim \frac{dM_{\mathrm{TRM}}}{dT} \qquad (3)$$

The distribution of magnetic anisotropy energies can be well fitted by a log-normal function

$$P = \frac{A}{D\sigma\sqrt{2\pi}} exp - \left[\frac{ln^2\left({}^{T}/_{\langle T_B\rangle}\right)}{2\sigma^2}\right] \qquad (4)$$

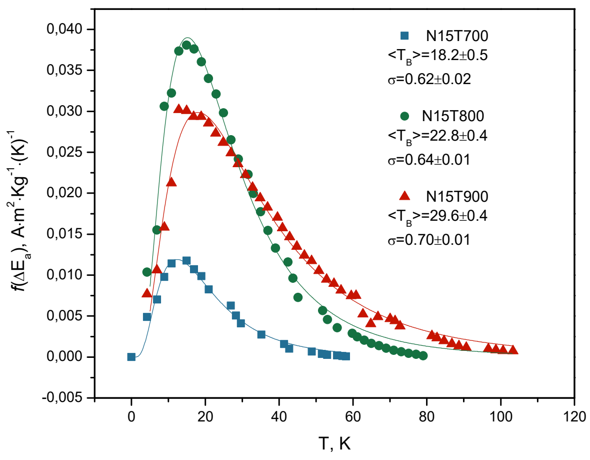

Figure S4. distribution of magnetic anisotropy energies (empty symbols) fitted by a log normal function (lines) for the samples N15T700 (squares), N15T800 (triangles), N15T900 (circles). Values of mean Blocking temperature (<TB>) and standard deviation (σ) are reported in the graph.

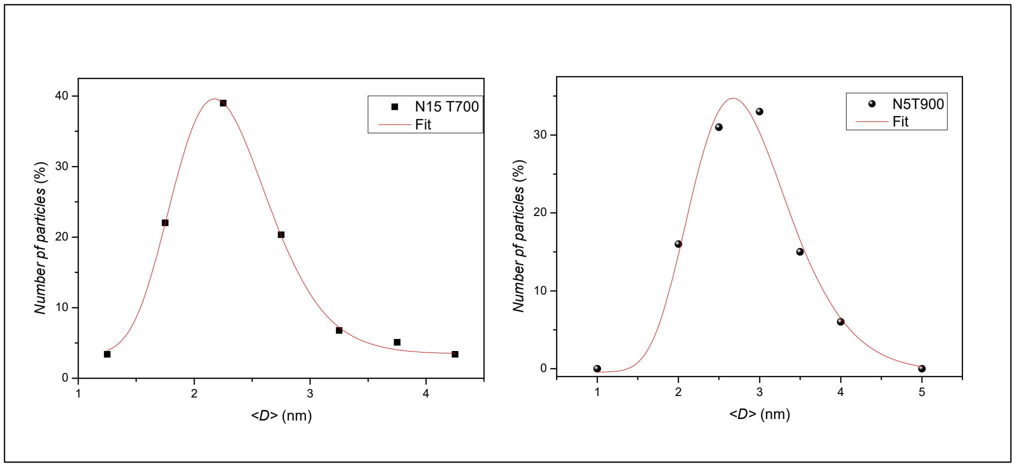

Figure S5. Particle size distribution extracted by TEM images of the sample N15T700 (left side) and N5T900 (right side).

The particle size distribution measured by TEM can be fitted by log-normal[6] function:

$$P = \frac{A}{D\sigma\sqrt{2\pi}}\, exp - \left[\frac{ln^2\left(D/\langle D_{TEM}\rangle\right)}{2\sigma^2}\right] \qquad (6)$$

where $\langle D_{TEM}\rangle$ is the median of the variable "diameter", often used to estimate the average diameter of nanoparticles 13–15 and σ the standard deviation of the natural logarithm of the variable D[6] (Table). To estimate the polydispersity of the sample with respect to the average particle size, an empirical parameter has been defined as:

$$PD = 100\, \times \frac{\sigma}{<D_{TEM}>} \qquad (7)$$

Table S1. Mean particle size (D_{TEM}) σ the standard deviation of the natural logarithm of the variable D and percentual polydispersity determined by equation (7).

	**N15T700**	**N15T900**
$\langle D_{TEM}\rangle$	2.3(1)	2.8 (1)
σ	0.19 (1)	0.22 (1)
PD(%)	8.26	7.85

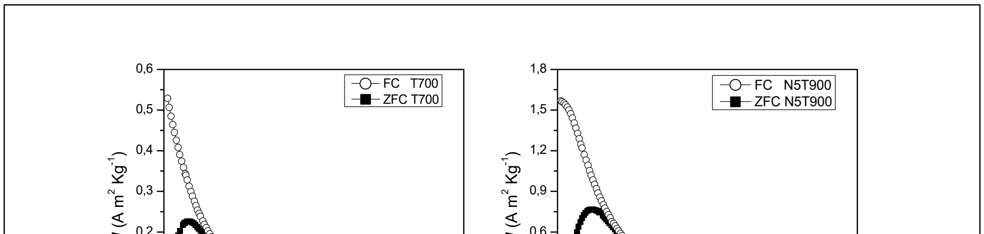

Figure S6. ZFC-FC curves for the N15T700 (a) and N5T900 (b).

Figure S6. shows ZFC FC curves measured with an external field of 2.5 mT for N15T700 and N5T900 samples. The behavior is typical of an assembly of superparamagnetic particles, whose moments block progressively with decreasing temperature, according to the distribution of their blocking temperatures. The continuous increase of M_{FC} with decreasing temperature gives a first qualitative hint about magnetic interparticle interactions, indicating that they are very weak in both samples. The larger T_{max} value for N5T900 (T_{max} = 41 K) with respect to N15T700 (T_{max} = 29 K) indicates a higher anisotropy in the first sample in agreement with the larger K_{eff} value [3].

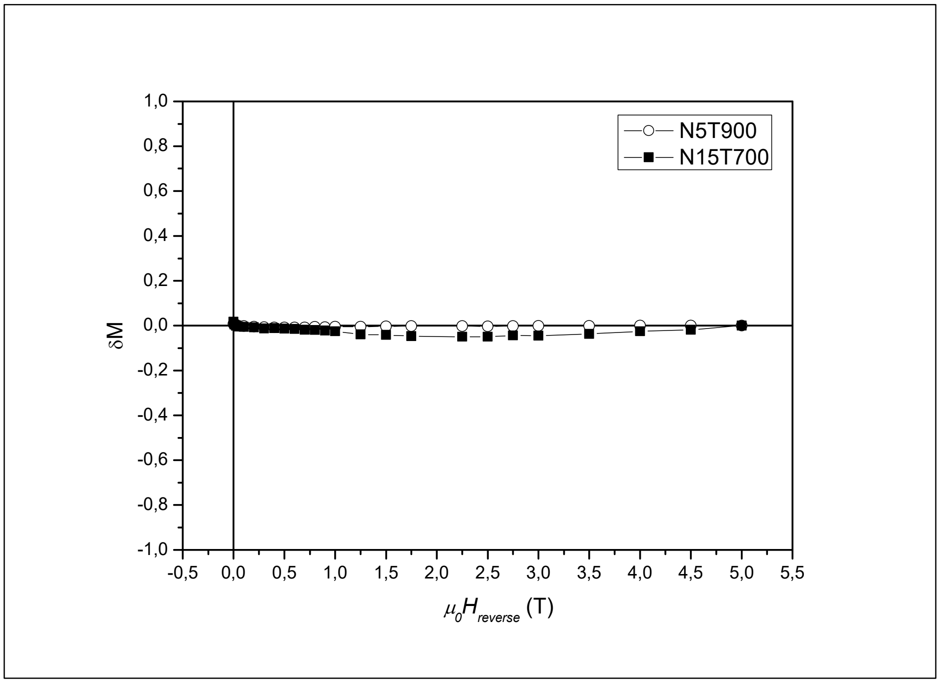

Figure S7. δM-plot at 5 K for N15T700 sample and reference sample N5T900 [3].

Magnetic interparticle interactions were investigated by measuring the isothermal remanent magnetization (IRM) and the direct current demagnetization (DCD) [5,6]. $M_{IRM}(H)$ was measured on the previously demagnetized sample applying a set of increasing fields (up to to 5 T), removing them and recording the remanent magnetization after each iteration. $M_{DCD}(H)$ was measured saturating first the sample, applying a set of increasing reverse fields and recording the remanent magnetization after each iteration. According to the Stoner–Wohlfarth model for an assembly of non-interacting randomly oriented MNPs with uniaxial anisotropy the two remanent magnetization curves are correlated by the following equation: $M_{DCD}(H) = 1 - 2 \cdot M_{IRM}(H)$. The effect of interparticle interactions can be evaluated by the Kelly equation [7]:

$$\Delta M(\mathrm{H}) = M_{\mathrm{DCD}}(\mathrm{H}) - 1 + 2 \cdot M_{\mathrm{IRM}}(H) \qquad (5)$$

where ΔM allows one to estimate the level of interactions. ΔM = 0 for an ideal situation with absence of interactions. A negative value indicates the predominance of dipolar interactions, whereas a positive value indicates the predominance of exchange interactions. Both samples show a negative deviation with intensity less than 0.05. These values can be considered within the experimental error, indicating that interparticle interaction in both samples can be considered negligible.

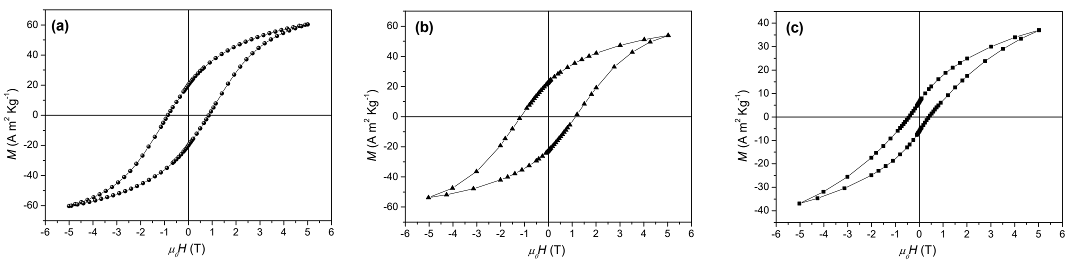

Figure S8. Hysteresis loop recorded at 5K for N15T900 (a), N15T800 (b) and N15T700 (c).

REFERENCES

[1] Cannas, C., Musinu, A., Peddis, D., and Piccaluga, G., **2006**. Synthesis and Characterization of CoFe2O4 Nanoparticles Dispersed in a Silica Matrix by a Sol-Gel Autocombustion Method. *Chemistry of Materials*, *18* (16), pp.3835–3842

[2] Muscas, G., Singh, G., Glomm, W.R., Mathieu, R., Kumar, P.A., Concas, G., Agostinelli, E., and Peddis, D., **2015**. Tuning the size and shape of oxide nanoparticles by controlling oxygen content in the reaction environment: Morphological analysis by aspect maps. *Chemistry of Materials*, *27* (6), pp.1982–1990.

[3] Peddis, D., Mansilla, M. V, Mørup, S., Cannas, C., Musinu, a, Piccaluga, G., D'Orazio, F., Lucari, F., and Fiorani, D., **2008**. Spin-canting and magnetic anisotropy in ultrasmall CoFe2O4 nanoparticles. *The Journal of Physical Chemistry. B*, *112* (29), pp.8507–8513.

[4] Peddis, D., Mansilla, M. V., Mørup, S., Cannas, C., Musinu, A., Piccaluga, G., D'Orazio, F., Lucari, F., and Fiorani, D., **2008**. Spin-Canting and Magnetic Anisotropy in Ultrasmall CoFe 2 O 4 Nanoparticles. *The Journal of Physical Chemistry B*, *112* (29), pp.8507–8513.

[5] Peddis, D., Jönsson, P.E., Laureti, S., and Varvaro, G., **2014**. Magnetic Interactions: A Tool to Modify the Magnetic Properties of Materials Based on Nanoparticles. in: Front. Nanosci., pp. 129–188.

[6] García-Otero, J., Porto, M., and Rivas, J., **2000**. Henkel plots of single-domain ferromagnetic particles. *Journal of Applied Physics*, *87* (10), pp.7376.

[7] Kelly, P.E., O'Grady, K., Mayo, P.L., and Chantrell, R.W., **1989**. Switching mechanisms in cobalt-phosphorus thin films. *IEEE Transactions on Magnetics*, *25* (5), pp.3881–3883.

© 2019 by the authors. Submitted for possible open access publication under the terms and conditions of the Creative Commons Attribution (CC BY) license (http://creativecommons.org/licenses/by/4.0/).
